# Simultaneous Gene Delivery and Tracking through Preparation of Photo-Luminescent Nanoparticles Based on Graphene Quantum Dots and Chimeric Peptides

**DOI:** 10.1038/s41598-017-09890-y

**Published:** 2017-08-25

**Authors:** Soroush Moasses Ghafary, Maryam Nikkhah, Shadie Hatamie, Saman Hosseinkhani

**Affiliations:** 1 Department of Nanobiothechnology, Faculty of Biological Sciences, Tarbiat Modares University, Tehran, Iran; 2 Institute for Nanoscience and Nanotechnology (INST), Sharif University of Technology, Tehran, Iran; 3 Department of Biochemistry, Faculty of Biological Sciences, Tarbiat Modares University, Tehran, Iran

## Abstract

Designing suitable nano-carriers for simultaneous gene delivery and tracking is in the research priorities of the molecular medicine. Non-toxic graphene quantum dots (GQDs) with two different (green and red) emission colors are synthesized by Hummer’s method and characterized by UV-Vis, Photoluminescence (PL), Fourier Transform Infrared (FTIR) and Raman spectroscopies, Atomic Force Microscopy (AFM), Scanning Electron Microscopy (SEM) and Transmission Electron Microscopy (TEM). The GQDs are conjugated with MPG-2H1 chimeric peptide and plasmid DNA (pDNA) by non-covalent interactions. Following conjugation, the average diameter of the prepared GQDs increased from 80 nm to 280 nm in complex structure, and the ζ-potential of the complex increased (from −36.87 to −2.56 mV). High transfection efficiency of the nano-carrier and results of confocal microscopy demonstrated that our construct can be considered as a nontoxic carrier with dual functions for gene delivery and nuclear targeting.

## Introduction

The emergence of gene therapy in the early 1990s caused a huge revolution in the molecular medicine field and created an effective strategy in the history of life sciences for the first time in order to treat genetic disorders such as cancer^1–3^. Generally, gene therapy refers to a series of therapeutic approaches in which a genetic material is transferred into specific cells of the patients’ body in order to repair and eliminate genetic defects. Although, many efforts have been carried out in this field, gene therapy is still in its early stages of development and even it has been accompanied with defeats in the past. Identification of therapeutic gene and its efficient delivery into the nucleus of the desired cells along with its tracking are key factors in a successful gene therapy process^3–5^. A suitable nano-carrier for gene therapy should have high efficiency, no cytotoxicity and immunogenicity, capable of carrying gene and protecting it against degradation, overcome biological barriers and gene delivery^6–10^. Nowadays, Non-viral vectors such as cationic lipids and polymers such as polypeptides have been raised as safe substitutes for viral vectors due to lack of harmful effects including cytotoxicity and immunogenicity. Hence, extensive efforts are in progress to find new non-viral nano-carriers for efficient delivery of therapeutic genes into desired cells. One of the most important carriers used in gene transfer into cells are fusion nano-peptides capable of condensing DNA, disrupting endosome membranes and enhancing the translocation of DNA towards nucleus^11, 12^. Chimeric peptide vectors used in this study are composed of three different motifs which are responsible for the three specific performances mentioned above in order to overcome the biological barriers in the path of gene transfer toward cellular nucleus. This peptide has been previously designed in our lab^8^. The first motif is a 16-mer peptide sequence containing positively charged amino acids such as lysine and arginine (ATPKKSTKKTPKKAKK) which are capable of binding and condensing of DNA molecule by electrostatic interactions. The main role of this motif is condensing and resizing of DNA molecule from micrometer to nanometer in order to facilitate its entry into the cells through the endocytosis route. The second motif (GALFLGFLGAAGSTMGA) has a key role in the disruption of endosomal membrane and the acceleration of endosomal escape of the designed complexes into the cytosol and therefore it’s protection against endosomal nucleases and peptidases. The peptides containing this motif are known as “fusion peptides”. The third motif is nuclear localization signal (NLS) (PKKKRKVA) which guides the desired peptide towards the nucleus through nuclear pore complexes (NPC)^7–9^.

Taking into consideration previous studies conducted on effective gene delivery, recent developments in the field of nanomedicine and the appearance of various tracking strategies, rendered possible the simultaneous tracking and imposing the therapeutic effects of genetic agents. A major challenge is design of a carrier which exhibits all of the criteria including, high transfection efficiency, cross cellular barriers, efficient cargo release, no toxicity or immune system stimulation, besides to the ability of tracking the delivered cargo. Therefore, the development of effective procedures with high precision and sensitivity can be promising step toward solving the current challenges of gene therapy. The fluorescent methods are appropriate strategies for rapid, careful and sensitive molecular imaging. In addition, these methods allow the multiplex detection of fluorescence probes with nanometer-scale resolution^13, 14^. Several studies shown wide applications of different fluorescent agents include organic fluorophores and fluorescence proteins for biological imaging and biomolecular tracking^14–17^. Organic fluorophores can penetrate biological structures and allow bio-labeling and subsequently tracking without interference in their biological functions^18^. But low brightness, least photo-stability, asymmetric and broad fluorescence spectrum, fluorescence blinking and difficult functionalization have limited their applications. Fluorescence proteins have some limitations compared to organic dyes due to their special photo-physical properties. For example, blinking, low brightness and photo-stability, limited colors and chemistry, fluorescent dependence on the cell type. In addition, aggregation of fluorescence proteins or generated free radicals with increasing cellular exposure time can induce cytotoxicity^19^. Quantum dots (QDs) as new fluorescence probes for biological imaging and tracking, have overcome the limitations of organic fluorophores and fluorescence proteins^18, 19^. Several studies have been conducted on gene delivery and tracked it by using QDs. Srinivasan *et al*. have designed nanostructures of pDNA labeled with phospholipid coated CdSe/ZnS QDs for tracking of gene. Their obtained complex has enabled gene transfection and cellular uptake monitoring^20^. Another study has investigated the conjugation of PEGylated QDs to both siRNA and F3 peptide and tracked the siRNA delivery by visualization of attached QDs under fluorescence microscope^21^. Despite the apparent advantages, one of the major barriers of semiconductor QDs is their cytotoxicity effects in biological systems, due to leakage of heavy metals. Therefore, their applications in molecular and cellular tracking have been limited. Various factors are important in determination of QDs toxicity level including size, charge, concentration, solubility in aqueous buffer, coating agents, etc. ref. 22. Despite numerous studies on the optimization of these factors for efficient applications of QDs in biological systems^20–23^, the toxicity of these nanocrystals is still a serious problem. Therefore, newly emerging quantum dots without toxic ions have been raised. The most important of those are graphene quantum dots (GQDs) which have attracted growing attention from many researchers^24–27^. GQDs have been designated as zero-dimensional graphene sheets (0D), with lateral dimensions less than 100 nanometers in one, two or few layers^26, 27^. The high photoluminescence and biocompatibility, optical and chemical stability, simple functionalization with biomolecules all together with non-cytotoxicity of these nanoparticles have proposed them as great promising luminescence probes in biological imaging and tracking^28–30^. In general, there are two strategies for the synthesis of GQDs: top-down and bottom-up. Top-down methods involve cutting graphene-larger materials to GQDs are widely used by researchers because of their simple methodology and high yield^31, 32^.

**Figure 1:**
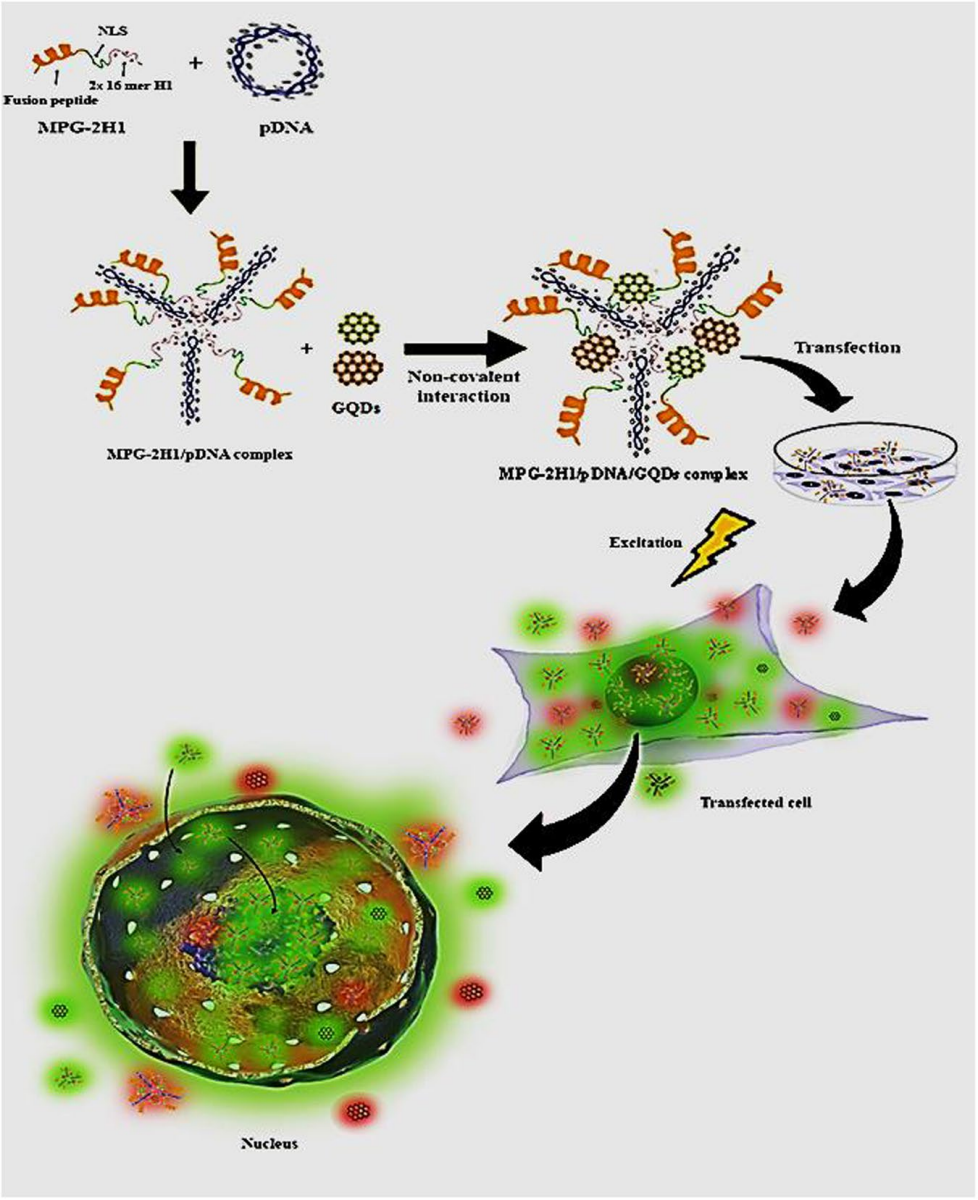
Schematic depiction of assembling MPG-2H1/pDNA/GQDs complexes through noncovalent interactions, transfection into the cells and excitation.

In this study, we have synthesized GQDs by Hummer’s method as one of the most known top-down methods, with two different sizes and hence two different (green and red) emission colors from a graphite source. As shown in Fig. 1, we have first prepared a complex of plasmid DNA (pDNA) and MPG-2H1 chimeric peptide, subsequently attached to GQDs, non-covalently. Finally, the prepared nanocomplexes were successfully transfected into HEK 293 T cell lines as a model to assess, qualitatively and quantitatively the efficiency of gene transfection, DNA delivery into the nucleus and tracking.

## Results and Discussion

### GQDs characterization

**Figure 2:**
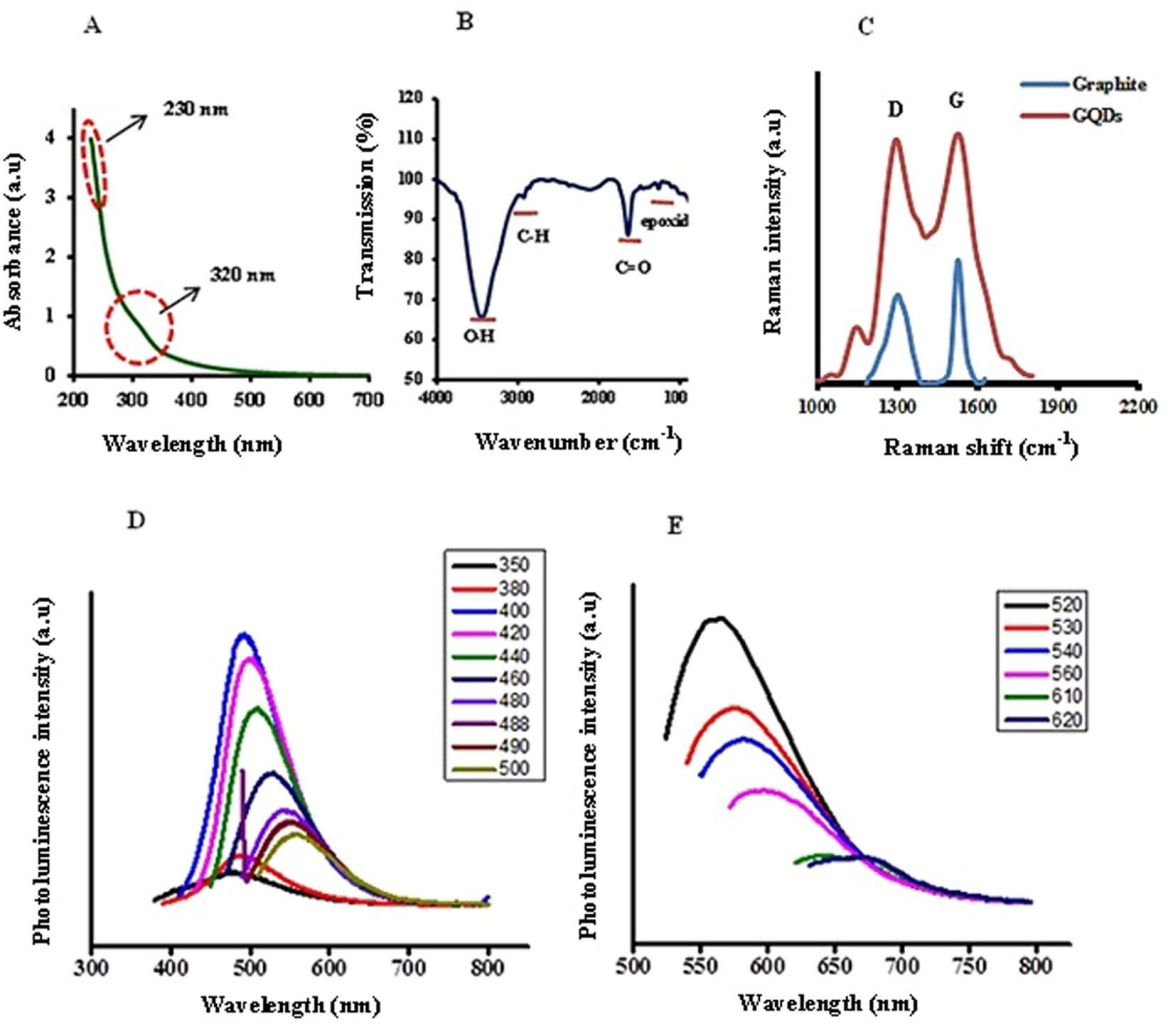
UV-Vis absorption spectrum (**A**) FTIR spectrum (**B**) Raman spectrum (**C**) and photoluminescence spectrums (**D** and **E**) of GQDs.

UV/Vis absorption spectroscopy of synthesized GQDs revealed an absorption shoulder in the visible region at 320 nm which is associated with n-π* transition and a strong absorbance at 230 nm attributed to π-π* transition (Fig. 2A), similar to previous reported studies for GQDs^33, 34^.The characteristic bands of surface functional groups on GQDs corresponding to epoxide, C=O, and O-H were detected by FTIR spectroscopy (Fig. 2B). Figure 2C shows Raman spectrum of graphite and GQDs. The two main peaks observed: the D-peak at 1301 cm^−1^ and the G-peak at 1524 cm^−1^. The relative intensity of the D-peak to the G-peak (I_D_/I_G_) for the GQDs and graphite is 0.97 and 0.71, respectively. High I_D_/I_G_ ratio of GQDs indicates higher disorders and structural defects in obtained GQDs^35^.

Several characteristics of these nanoparticles including quantum confinement and edge effects, surface defects and free zig-zag sites in the GQDs have been considered as the source of their fluorescence^34, 36, 37^. The PL spectrum of the synthesized GQDs exhibited excitation-dependent fluorescence. When the excitation wavelength increased from 350 to 500 nm and from 520 to 620 nm, the maximum of emission peak shifted from 500 to 570 nm and from 575 to 675 nm, respectively. As well as, by changing the excitation wavelength, PL intensity changed too; a maximum emission was observed at 500 nm when excited at 400 nm, whereas when excited at 520 nm an emission peak was observed at 575 nm (Fig. 2D and E).

**Figure 3:**
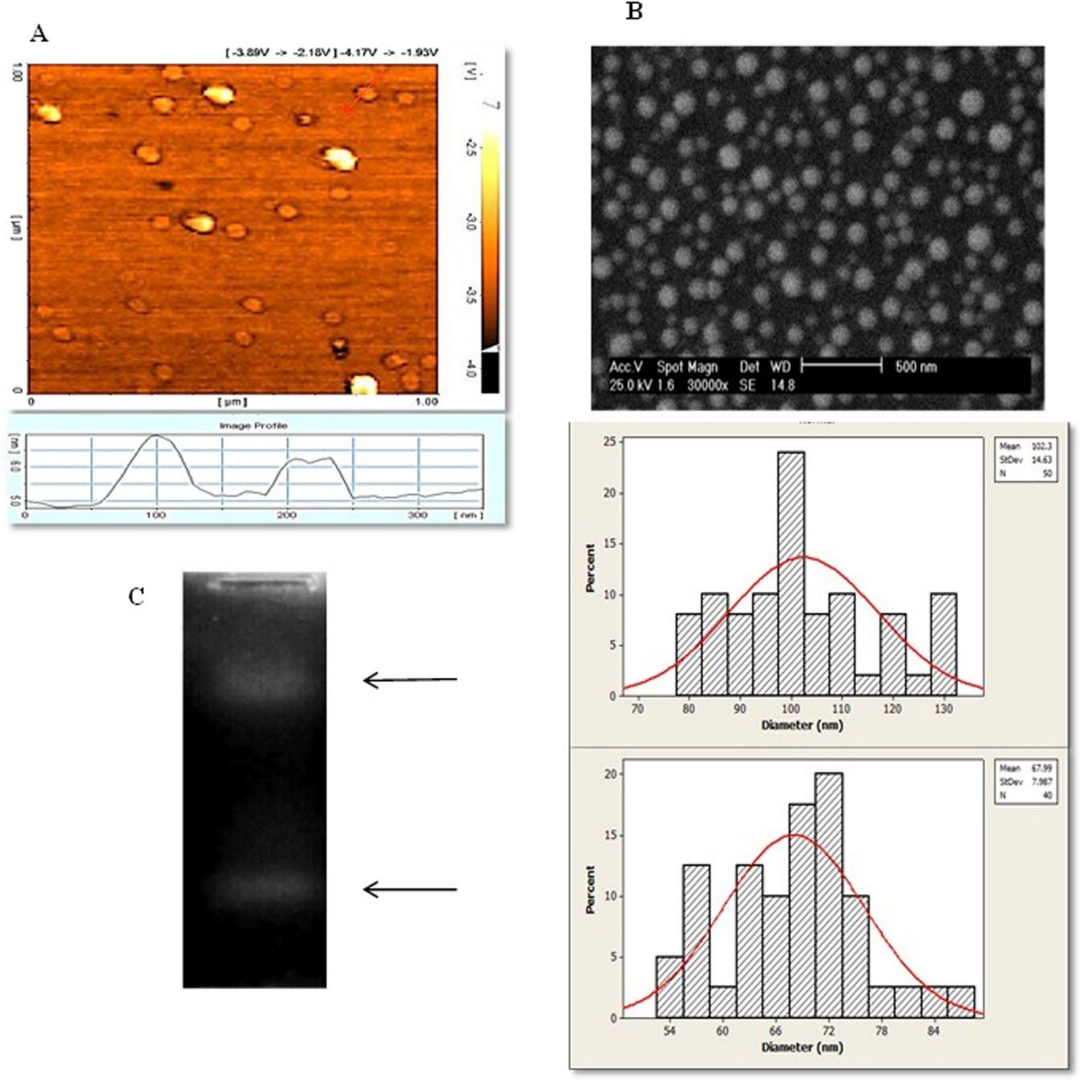
AFM topography image of GQDs (**A**), the bottom image shows height distribution of GQDs. SEM image and size distribution analysis of GQDs (**B**). Separation of two populations of free-GQDs by electrophoresis on 1% agarose gel (**C**).

Figure 3A shows a typical AFM image of the GQDs. As shown in the right bottom image in Fig. 3A, the average topographic height of GQDs is <1 nm (about 0.7 nm), indicating that the synthesized GQDs consist of a single graphene layer^38^. AFM and SEM (Fig. 3B) characterization revealed that the average size of GQDs is about 80 nm. The average size of small and large GQDs in SEM image, determined using imagJ software, is about 68 nm and 100 nm, respectively. Normal distribution curve and histogram chart were plot using Minitab 16 software. The synthesized GQDs showed two distinct bands on agarose electrophoresis gel (Fig. 3C), which is in accordance with the AFM data and SEM size distribution analysis of GQDs and demonstrates that two-color emitter GQDs were successfully obtained.

### MPG-2H1 peptide expression

**Figure 4:**
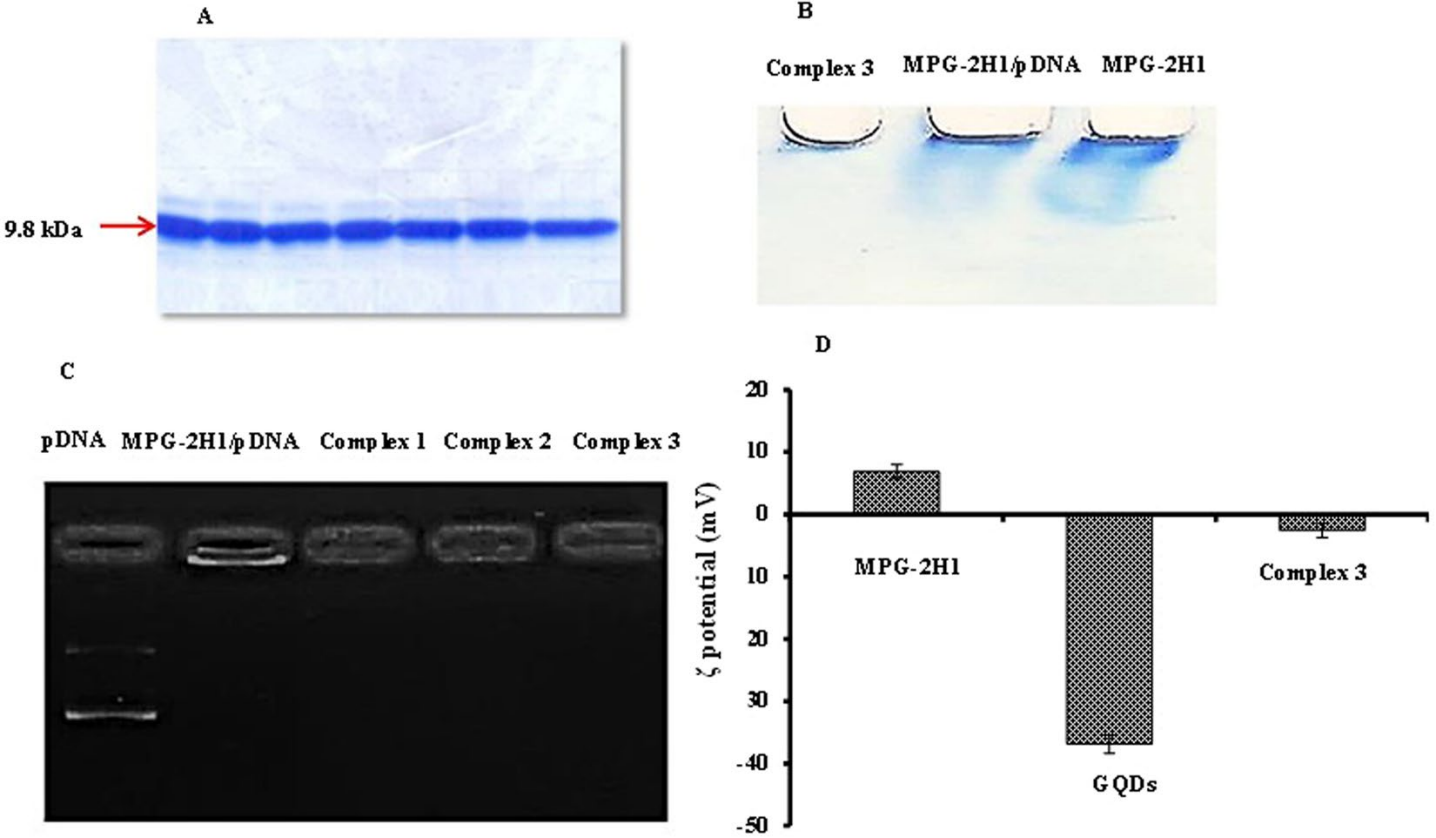
The coomassie blue stained purified peptide in 15% SDS-PAGE (MW.14 kDa) (**A**). Native acrylamide gel retardation (PAGE 15%) assay of MPG-2H1, MPG-2H1/pDNA and MPG-2H1/pDNA/GQDs complex (**B**). Cropped agarose gel electrogram (1%) pDNA retardation assay (**C**). ζ-potential of MPG-2H1 peptide, GQDs, and MPG-2H1/pDNA/GQDs complex 3 (**D**), error bars denote the standard deviation.

There is a His-tag tail in the C-terminal of MPG-2H1 peptide which allows purification using Ni-NTA resin. As previously described, the pET28a plasmid harboring the gene encoding the fusion peptide is expressed in *E*. *coli* C41 (DE3) plysS. Then, purification was done by using Ni-NTA resin under urea gradient. Figure 4A shows the purified peptide as a single band in 15% SDS-PAGE gel.

### Investigation of the GQDs/MPG-2H1 peptide/pDNA complex formation

Preliminary investigation of the complex formation was performed by gel retardation assay (Fig. 4B and C). Acrylamide-based gel retardation demonstrated that the dual and ternary stable complexes including MPG-2H1 peptide/pDNA and MPG-2H1peptide/pDNA/GQDs were successfully formed, based on the values inserted in Table 1. As shown in Fig. 4B free MPG-2H1 peptides travel a longer distance in the gel when compared to MPG-2H1/pDNA mixture, whereas the MPG-2H1/pDNA/GQDs complexes travel in the opposite direction.

**Table 1:** The utilized values of GQDs, MPG-2H1 peptide and pDNA for preparation of the complexes.

	Complex 1	Complex 2	Complex 3	Complex 4	Complex 5
GQDs (nmol)	9.6 × 10^−2^	1.44 × 10^−1^	1.6 × 10^−1^	1.6 × 10^−1^	2.4 × 10^−1^
MPG-2H1 peptide (µg)	24.3	24.3	24.3	30	24.3
pDNA (µg)	1.0	1.0	1.0	1.0	1.0

We also performed agarose-based gel retardation assay followed by ethidium bromide staining to confirm pDNA attachment to the complexes (Figs 4C and S1) and the results showed that the mobility of pDNA decreased when conjugated to the peptide. The pDNA band was remained in the well due to charge neutralization of the MPG-2H1/pDNA complex.

The positively charged MPG-2H1 peptide allows effective attachment of the negatively charged pDNA, however, by decorating this complex with GQDs the pDNA band was not observed on the agarose gel, due to the possible quenching of ethidium bromide by GQDs. The average ζ-potential of the MPG-2H1 peptide, free GQDs and MPG-2H1 peptide/pDNA/GQDs complex was +6.85, −36.87 mv and −2.56 mv, respectively (Fig. 4D). The change in the ζ-potential of the complex reveals that electrostatic interactions have been established.

**Figure 5:**
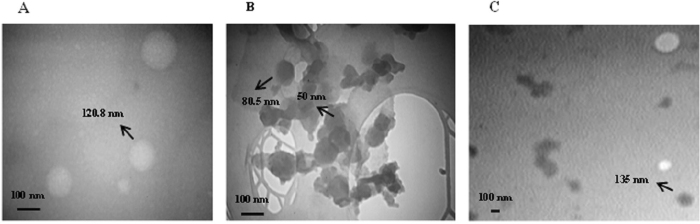
TEM image of the negatively stained targeted MPG-2H1/pDNA complex^7^ (**A**) GQDs (**B**) and MPG-2H1/pDNA/GQDs complex (**C**).

The morphology and size distribution of both GQDs and complexes were determined by TEM. Results show that the average size of MPG-2H1/pDNA complexes is about 200 nm (Fig. 5A)^7^. The average diameter of GQDs is about 80 nm (Fig. 5B) and the average size of obtained MPG-2H1/pDNA/GQDs complexes is between 135–280 nm (Fig. 5C). The synthesized GQDs had a stacked structure where they were small round layers placed on a larger thinner layer as previously shown once for GQDs^39^.

**Figure 6:**
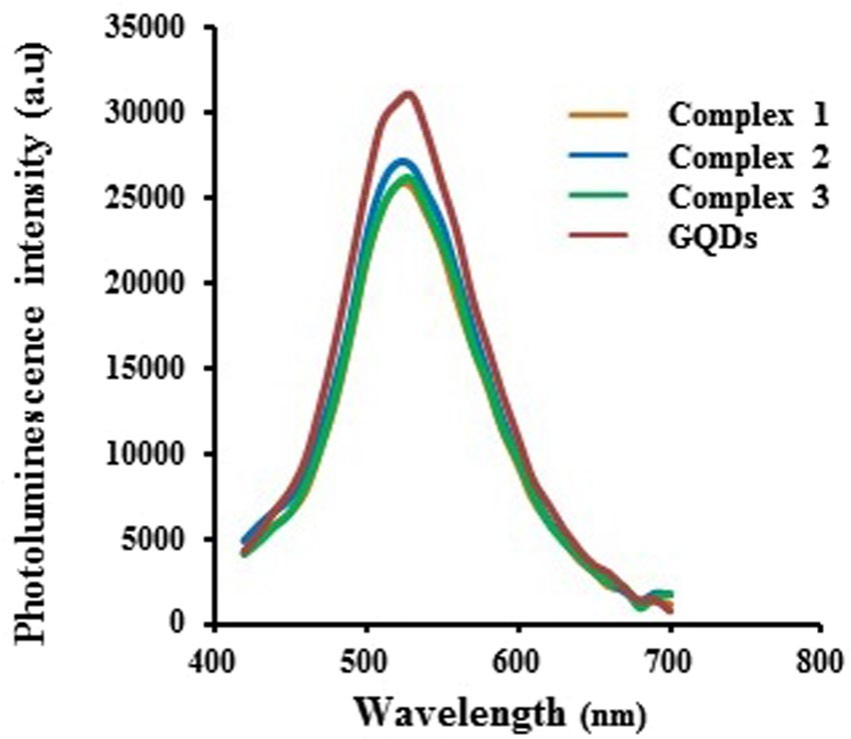
Emission spectrum of GQDs, complex 1, 2 and 3 (excitation at 400 nm).

**Figure 7:**
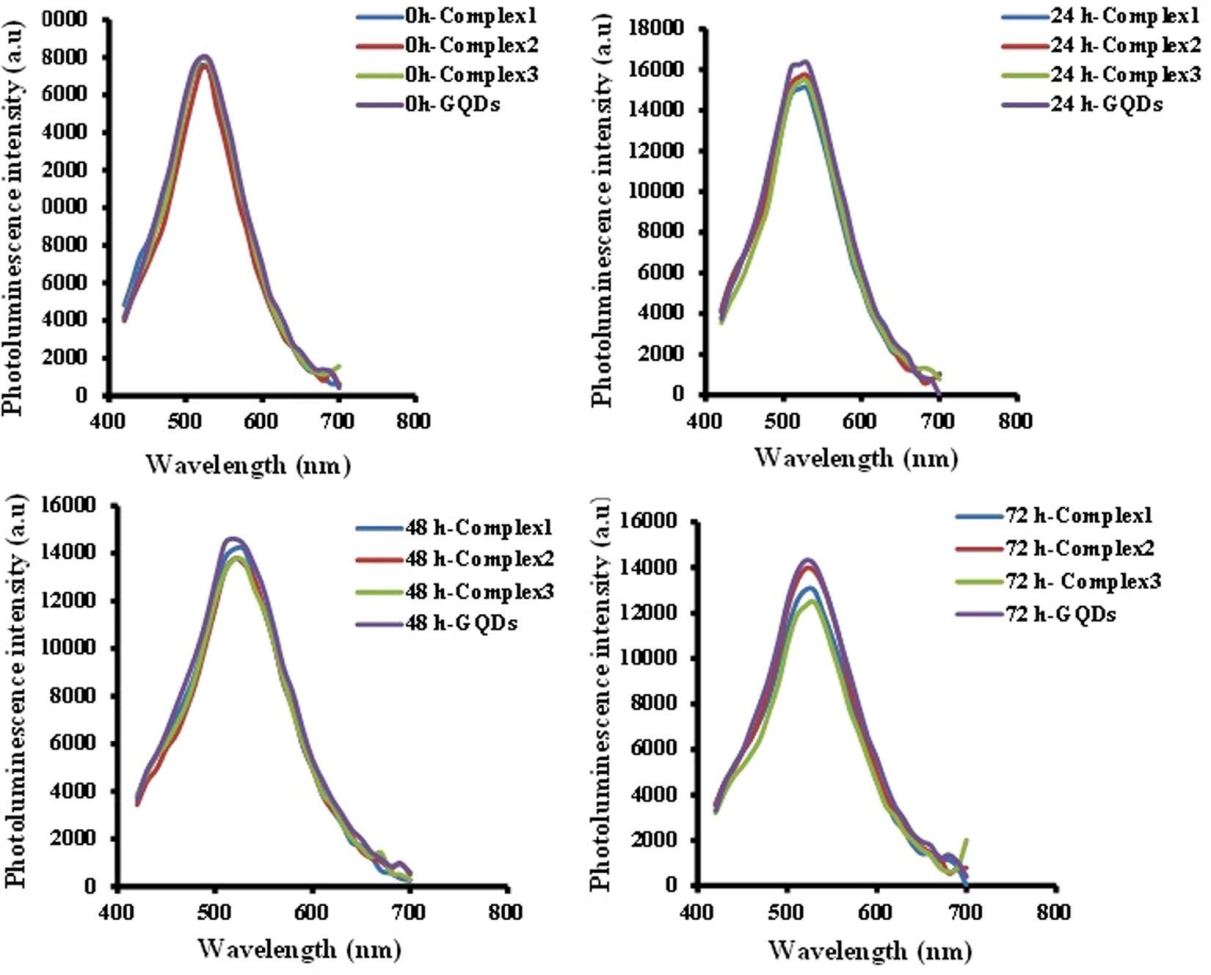
Stability assay of GQDs and complexes at different time intervals (24, 48 and 72 h).

The investigation of PL properties of MPG-2H1/pDNA/GQDs complexes in comparison to free GQDs revealed that the attachment of MPG-2H1/pDNA complex to GQDs doesn’t have any significant effects on the GQDs’ PL intensity (Fig. 6). In addition, the fluorescence stability of the free GQDs and complexes was assessed in 24 h, 48 h and 72 h. The results showed that likewise, there is no significant difference between their fluorescence intensity and after each 24 h just a negligible decrease in both of GQDs and complexes was observed (Fig. 7).

### Evaluation of nanoparticles toxicity

**Figure 8:**
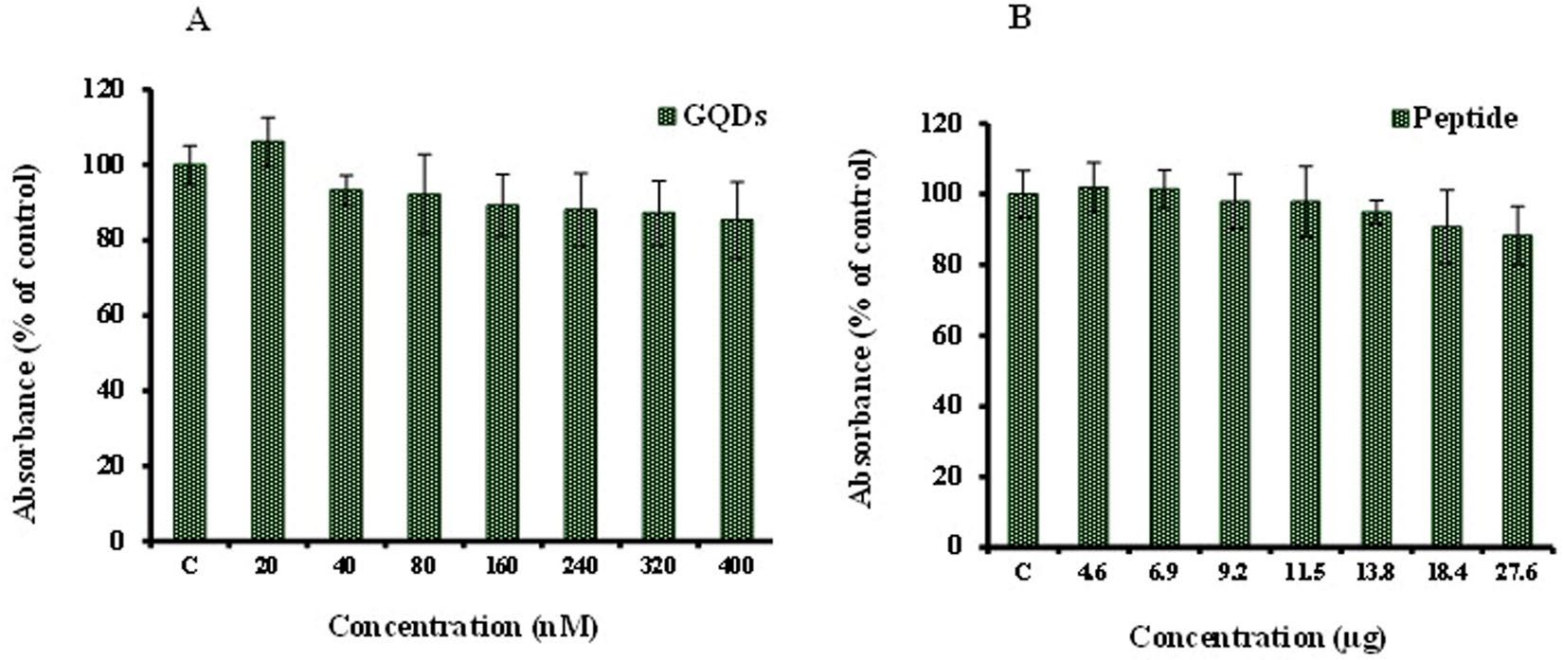
Cell viability assays of GQDs (**A**) and peptide (**B**) for 48 h. C shows control cells incubated in cell culture medium without GQDs or peptides.

The cytotoxicity of different concentrations of MPG-2H1 peptides and synthesized GQDs after 48 h was investigated by MTT assay. The results showed that the assessed concentrations of GQDs didn’t show significant cytotoxicity in HEK 293 T cells after 48 h (up to 400 nM) as well as MPG-2H1 chimeric peptide up to a concentration of 27.6 µg (Fig. 8). Similar to our results several studies have confirmed the non-cytotoxicity of graphene quantum dots^40–43^.

### Cellular uptake imaging

**Figure 9:**
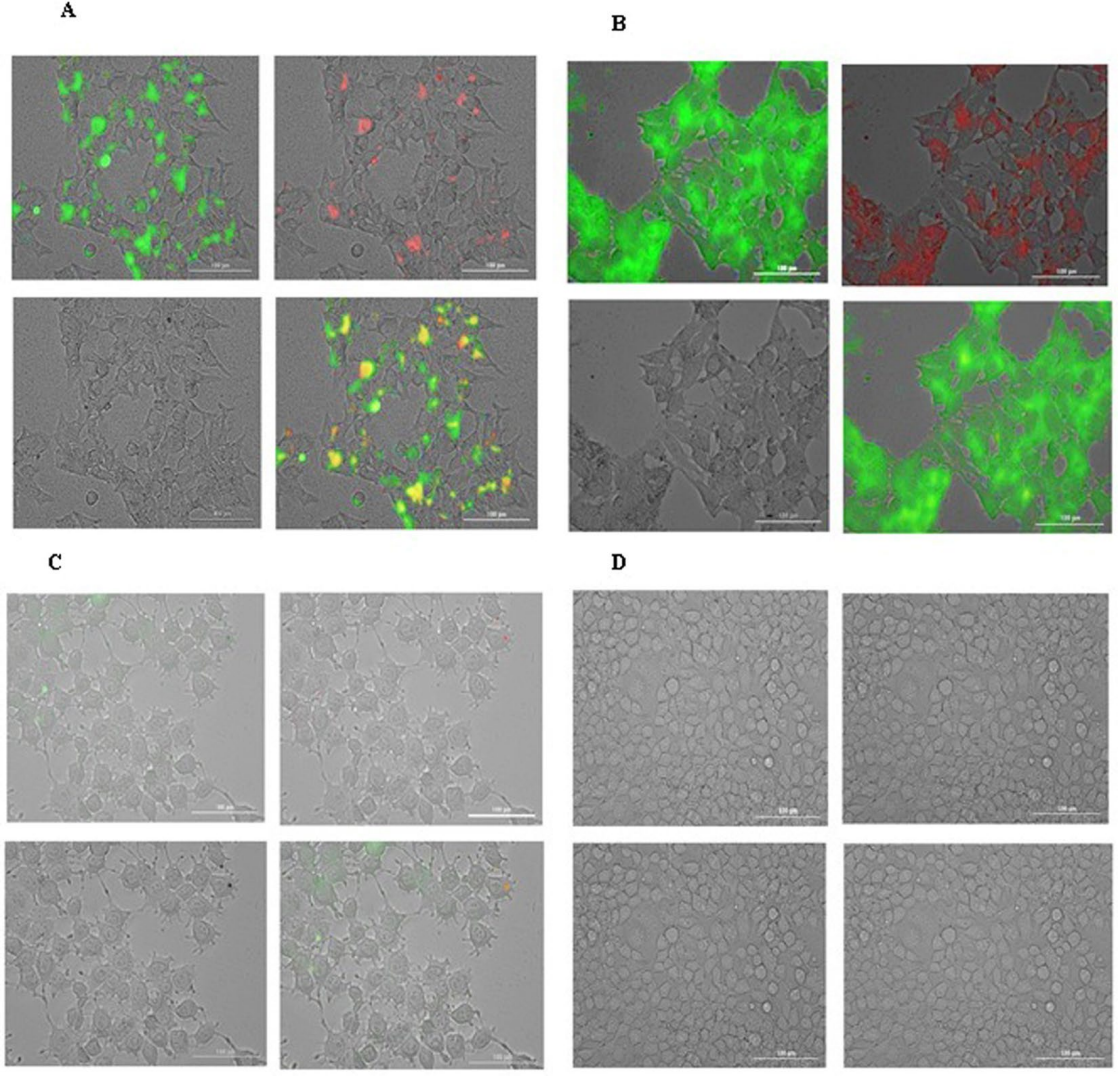
Fluorescence microscopy images of transfected cells with complex 1 (**A**), complex 3 (**B**), GQDs (**C**) and control cells (**D**) are shown in 4 different panel. In each panel: left top is excited by 469 nm and detected in 525 emission wavelength, right top is excited by 585 nm and detected in 647 emission wavelength, left bottom shows bright field and overlay is indicated in right bottom.

To determine qualitative uptake of complexes, HEK 293 T cells were exposed to GQDs and complexes in different concentrations of MPG-2H1 peptide, pDNA and GQDs and then analyzed by Cytation^TM^ 3 multi-mode detection system (Fig. 9). Control cells did not show any fluorescence (Fig. 9D), and only a small fraction of the free GQDs entered the cells (Fig. 9C). However, the complexes were able to enter the cells (Fig. 9A and B). In order to detect free and conjugated GQDs, two different wavelengths [(ex:469, em:525) and (ex:585, em:647)] were used (left top and right top images, respectively, in each four part of Fig. 9). Complex 3 revealed the most internalization into the cells and in the next lower level were complex 2 (data not shown) and complex 1, respectively (Fig. 9A and B).

Interestingly, cells transfected with the complexes displayed significantly both green and red fluorescence. These results confirmed the already obtained results based on existence of two populations of GQDs prepared from our synthesis procedure.

Generally, the results demonstrated that the free-GQDs can’t significantly enter the cells and noncovalent association with MPG-2H1 peptide can increase the rate and efficiency of GQDs uptake into the living cells. According to our results after 6 h complexes started to enter the cells and increased after 12 h (data not shown) to reach its maximum after 24 h. So far, GQDs have been successfully used for tracking breast carcinoma cells (T47D), neural stem cells (NScs), polyclonal plasmablastic cells (PPCs) and cardiogenic progenitor cells (CPCs) within 24 h^43^. In a study conducted by Zhang *et al*., the results obtained from confocal fluorescence microscopy images revealed that GQDs entered the cytoplasm but they had relatively weak PL in the nucleus^43^. However, in our study free GQDs couldn’t enter the cells, significantly; probably due to the high negative charge on their surface. In addition, in another study, Su *et al*. have designed peptide nanofibers decorated with GQDs by noncovalent interactions for imaging of tumor cells. Similar to our results, their constructs could easily enter alive cells^44^, however free-GQDs did not show significant entry into the cells.

**Figure 10:**
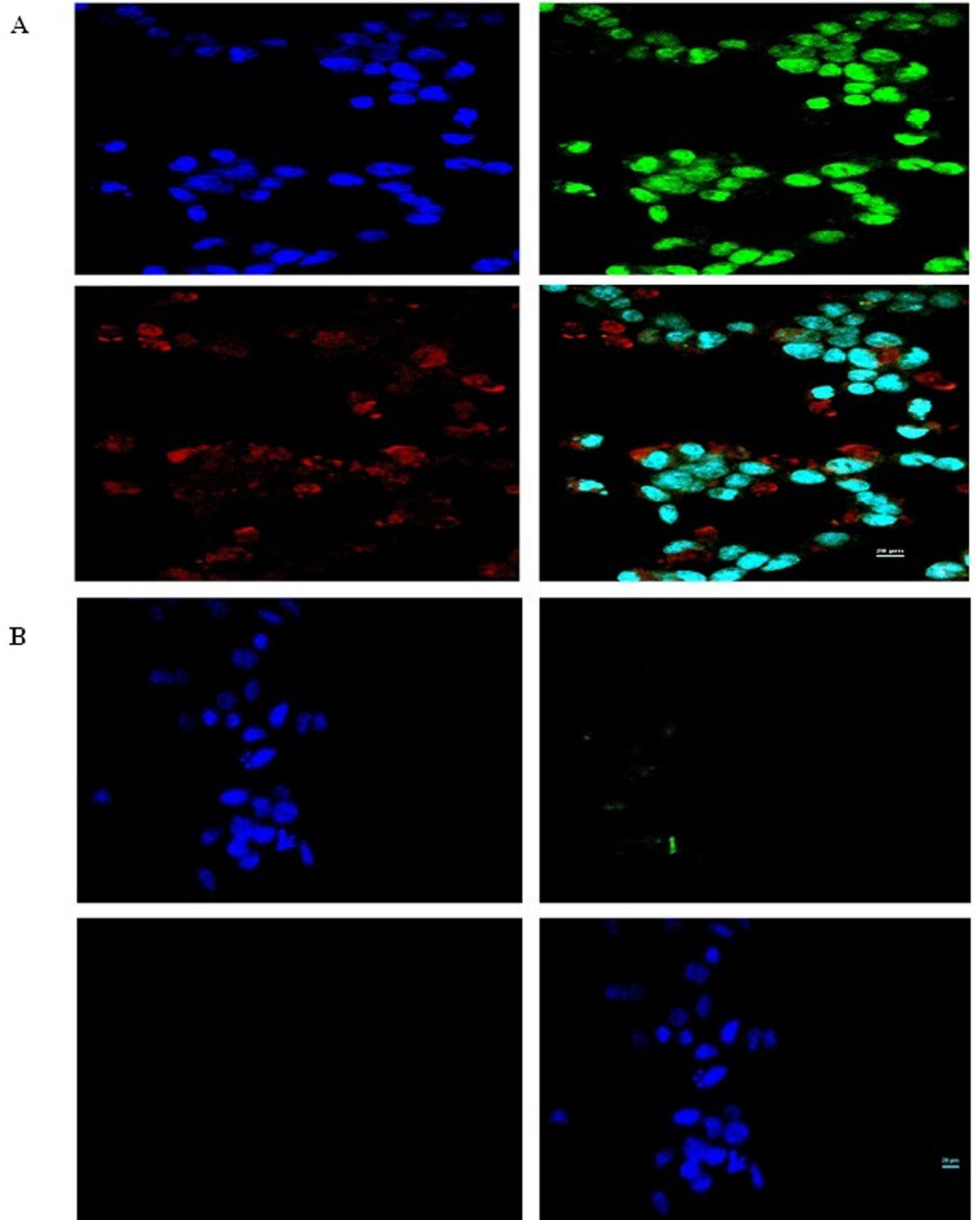
Confocal microscopy images of transfected cells with complex 3 (**A**) and GQDs (**B**). Blue color show DAPI nucleir stain, green and red show GQDs. Four images of each part are: excited by 408 nm for detecting DAPI nucleir stain (left top), excited by 457 nm for detecting green GQDs (right top), excited by 642 nm for detecting red GQDs (left bottom) and overlay (right bottom).

The results obtained from confocal microscopy confirmed previous imaging results and revealed significant difference between free GQDs transfected cells and the complexes transfected cells (Fig. 10). Interestingly, green light emitting GQDs complexed with MPG-2H1/pDNA bypassed nuclear pore complex via the NLS motif of MPG-2H1 to guide the pDNA toward the nucleus. However, only a small fraction of red GQDs could escape the nuclear membrane, probably due to their larger size, and most of them were detained surrounding the nucleus. It might be concluded that the green GQDs attached to MPG-2H1-psiCHECK could enter the nucleus while bigger red GQDs couldn’t enter the nucleus as good as the green ones. Probably along with the cell division, higher amounts of red fluorescence GQDs could enter into the nucleus of daughter cells. In this study, GQDs and NLS chimeric peptide (MPG-2H1) were attached by simple noncovalent interactions however, in a recent report, Maity *et al*. revealed covalently attachment of core-shell CdSe-ZnS QDs with NLS peptide (PKKKRKVKAA) using an approach contains three tedious and time*-*consuming decoration stages^45^.

### Cell Transfection

**Figure 11:**
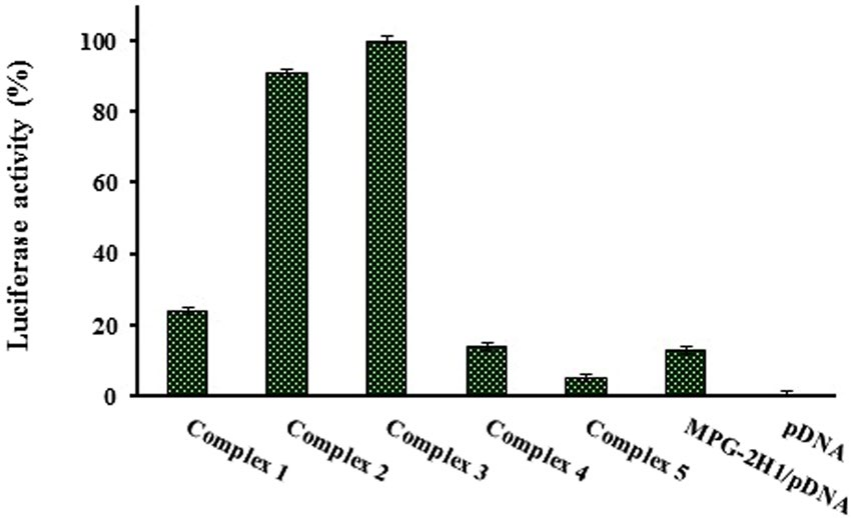
Transfection of psiCHEK plasmid harboring luciferase gene using different complexes of MPG-2H1/pDNA/GQDs and MPG-2H1 peptide/pDNA. Luciferase activity is indicator of transfection efficiency.

According to the results obtained from the gel retardation, cytotoxicity assays and bioimaging, the best ratios of GQDs, peptide and pDNA were obtained (Table 1). Luciferase activity was measured as already described. Branch PEI (25 kD) with N/P ratio of 8 was used for verification of the transfection efficiency of the complexes. The highest transfection efficiency was obtained for complex 3 consisting of 1.0 µg pDNA, 24.3 µg MPG-2H1 peptide and 1.6 × 10^−1^ nmol GQDs. It worth mentioning that by increasing the GQDs to the amount of 1.6 × 10^−1^ nmol in to the constant values of 24.3 µg MPG-2H1 peptide and 1.0 µg pDNA (N/P ratio = 18) the efficiency of transfection was increased. However, the further increase of GQDs or peptide concentrations (complex 4 and complex 5), resulted in lower transfection efficiency (Fig. 11). Therefore, all of the three components of our constructs were conjugated in optimal proportions in complex 3. Interestingly, complex 3 showed 5-fold increase in transfection efficiency than the MPG-2H1 peptide/pDNA complex (Fig. 11).

**Figure 12:**
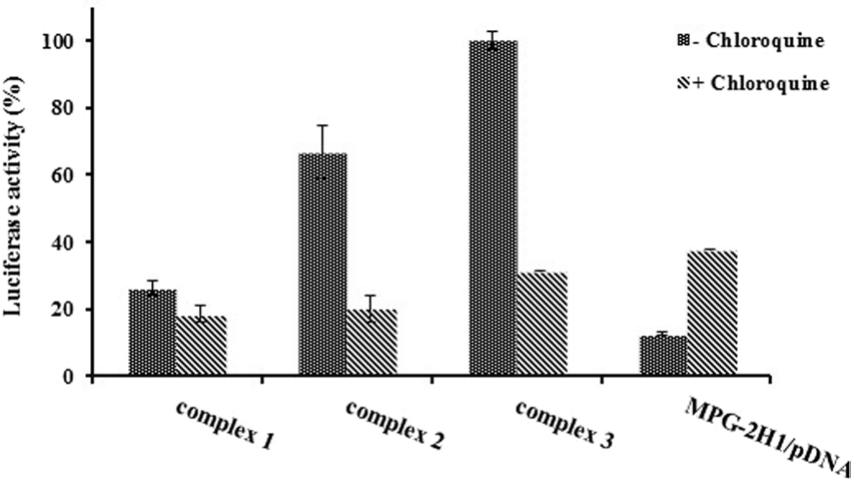
Effect of chloroquine on transfection efficiency of psiCHEK plasmid harboring luciferase gene using different complexes of MPG-2H1/pDNA/GQDs and MPG-2H1 peptide/pDNA. Luciferase activity is indicator of transfection efficiency.

Unexpectedly, the obtained results revealed that chloroquine as a lysosomotropic agent^46–48^, decreased the efficiency of transfection of MPG-2H1/pDNA/GQDs complexes, on the contrary, it promoted the transfection of MPG-2H1/pDNA complexes (Fig. 12). It might be concluded from special chemical structures of chloroquine and GQDs nanoparticles that it interfere with π-π stacking and electrostatic interactions between aromatic rings and their functional groups, respectively. Therewith, the complexes may become unstable and chloroquine competes with MPG-2H1 peptide for interaction with pDNA and therefore decreases gene expression.

### Fluorimetric assay

**Figure 13:**
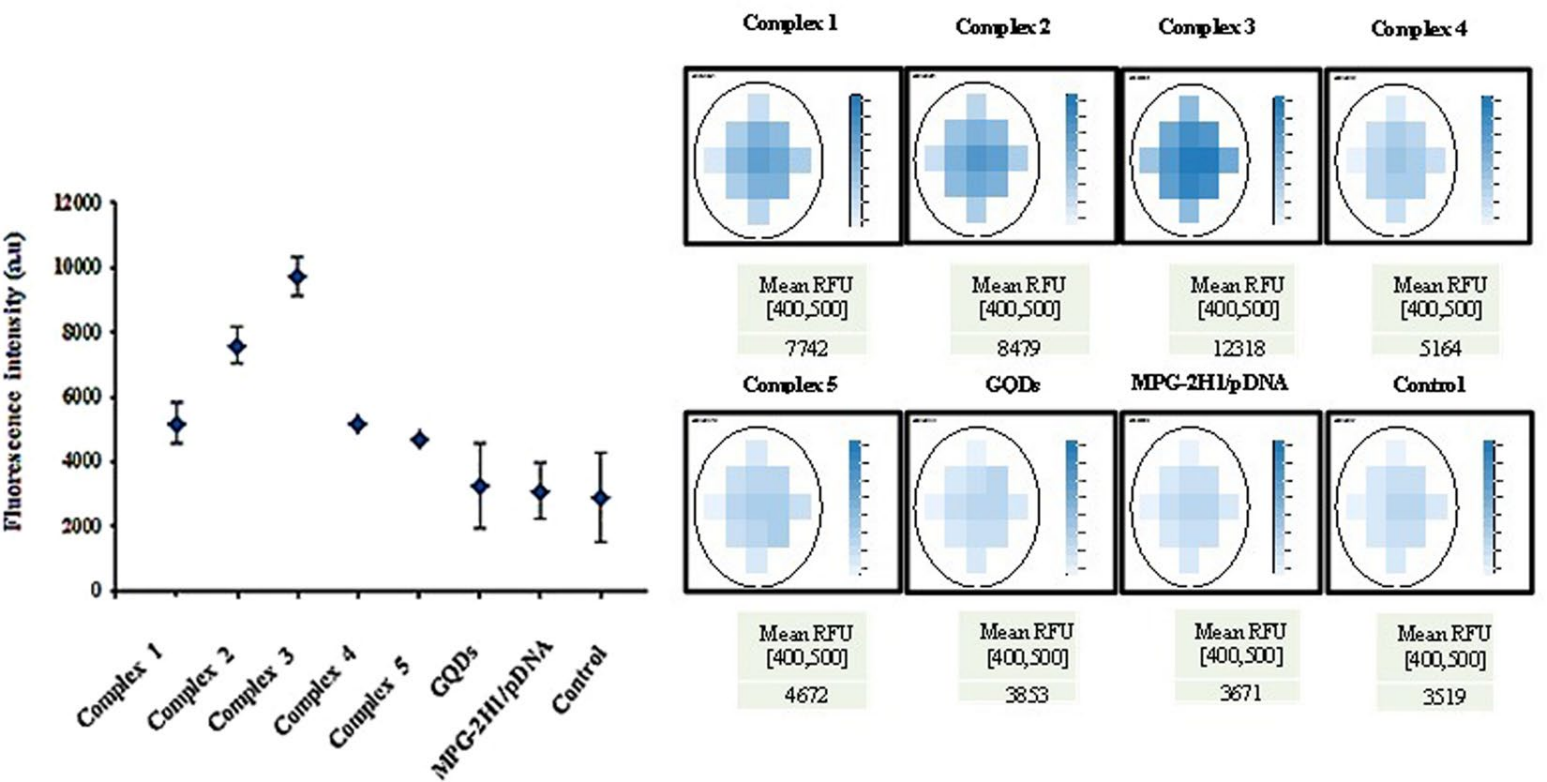
Fluorimetric assay of transfected cells with complexes 1 to 5, GQDs, MPG-2H1/pDNA complex and control cells using scanning area mode.

The fluorimetric data confirmed the previous results as the cells transfected with complex 3 showed the highest fluorescence signal, whereas those transfected with either complex 2, complex 1, complex 4 and complex 5 are placed respectively in the lower ranks. The cells transfected with GQDs didn’t show significant difference with the cells transfected with MPG-2H1/pDNA complex or non-transfected control cells. Typical data and selected wells are shown in Fig. 13, as well as the average fluorescence of each well was plotted in a dot plot.

The results obtained from bioimaging, cell transfection and fluorimetric assays indicate that using specific ratios of GQDs to MPG-2H1/pDNA, complexes can make the most suitable nano-carriers with dual ability of tracking and delivering gene into living cells and overcoming cellular trafficking, efficiently.

In this study, we succeeded in nuclear targeting and gene tracking, simultaneously, for the first time, by simple noncovalent interactions between GQDs and chimeric peptide/pDNA complexes.

## Conclusions

In conclusion, according to the results presented in this manuscript, it can be concluded that a novel approach for concomitant delivery of pDNA and GQDs with two different green and red emissions have been performed through non-covalent attachment to a reported chimeric peptide with ability of DNA packaging, endosomal escape and cellular nuclear targeting. GQDs could allow efficient tracking and enhance internalization of the plasmid harboring firefly luciferase gene into HEK 293 T cells. Our designed nano-carrier can be considered a promising vector for enhanced nuclear internalization and tracking of pDNA *in vitro* and *in vivo*.

## Materials and methods

### Reagents

*E*. *coli* C41 (DE3) pLysS was obtained from Novagen. pET28a expression vector was purchased from Invitrogen. Fetal bovine serum (FBS) and Dulbecco’s Modified Eagle’s Medium F12 (DMEM F12) were purchased from Gibco, plasmid extraction kit and Ni-NTA-Sepharose column from Qiagen. IPTG obtained from Vivantis. Luciferin was purchased from Resem (the Netherlands). Adenosine triphosphate (ATP) and dialysis bag (2.5 KDa) were purchased from Sigma. MTT 3-(4,5-s-dimethylthiazol-2-yl)-2,5-diphenyl tetrazolium bromide was obtained from Sigma–Aldrich. Graphite powder (mesh~200 µm), KMnO_4_, NaNO_3_, H_2_O_2_, Dimethylformamide (DMF) were purchased from Chemlab. Sulfuric acid (98%) was obtained from Merck.

### Over expression and purification of MPG-2H1 chimeric peptide

In order to express the designed chimeric peptide, first the expression vector (pET28a) was transformed into *E*. *coli* C41 (DE3) pLysS. Then 10 ml of LB medium containing 50 mM kanamycin was incubated with a fresh bacterial colony harboring the expression plasmid and grown at 37 °C overnight under shaking at 200 rpm. 1 ml of pre-cultured bacteria was used to inoculate 250 ml 2xyt medium and grown at 37 °C under vigorous shaking until the OD_600_ reached 0.4. The mixture was induced by IPTG to a final concentration of 0.2 mM and incubated at 37 °C for 4 h in 200 rpm shaking. The cells were harvested by centrifugation at 6000 rpm for 20 min at 4 °C. The cell pellet was resuspended in lysis buffer [20 mM Tris-Base, 500 mM NaCl, 8 M urea, 5 mM imidazole (pH = 11)] and incubated at 20 °C for 1 h with shaking at 200 rpm. Centrifugation was done twice in 6000 and 12000 rpm at 4 °C for 20 min to pellet insoluble fractions. Then the supernatant was loaded onto Ni-NTA-Sepharose column and incubated for 1 h at room temperature. Then the column was washed by eight washing buffers with increasing imidazole and decreasing urea concentration [20 mM Tris-Base, 1.0 M NaCl, 7.0 M, 6.0 M, 4.0 M, 2.0 M and 0 M urea, 20 mM, 40 mM, 60 mM and 70 mM imidazole, (pH = 8)]. Urea gradient was applied to gradual elimination urea and subsequently refold MPG-2H1 peptide slowly. The desired peptide was eluted from the column by elution buffer [20 mM Tris-Base, 500 mM NaCl, and 250 mM imidazole; (pH = 8)]. After observing the peptide bands on 15% sodium dodecyl sulfate-polyacrylamide gel electrophoresis (SDS-PAGE), Purified peptide was desalted by dialysis (cut-off: 2.5 KD) against PBS buffer, pH = 7.4 at 4 °C for 48 h. Dialysis buffer was exchanged every 6 h. Finally, Peptide stocked and stored at −20 °C for subsequent steps.

### Synthesis of graphene oxide

Graphene oxide was synthesized using a modified Hummer’s method as reported previously^49^. Graphite powder [0.5 g (≤20 µm, Fluka)] was dispersed in 23 ml sulfuric acid at 0 °C. Then, 0.5 g NaNO_3_ and 3 g KMnO_4_ was added into the mixture and solution was stirred for 3 h at 35 °C. Finally 40 ml deionized (DI) water was added and followed by addition of a solution of 100 ml DI water and 3 ml H_2_O_2_ (30%). The yellow color graphite oxide suspension was filtered and washed with HCL and water to remove extra ions (with volume ratio 1:10 for HCl to DI water). Washing process using DI water was repeated several times to balance the pH around 5, then the samples was sonicated (100 kHz, 120 W) for 40 min to exafoliate the graphene oxide sheets. the suspention was centrifuged for (3500 rpm, 30 min) to remove unexafoliated sheet of graphite oxide^50–52^.

### Preparation of GQDs

GO (270 mg) was dissolved in DMF (20 ml) and the solution was sonicated for 30 min (120 W, 100 kHz). Then the GO/DMF mixture was transferred to a poly (tetrafluoroethylene) (Teflon)-lined autoclave (30 ml) and heated at 200 °C for 5 h. When the reaction was completed, the reactors were cooled to room temperature. The reaction products includes yellow-brown supernatant and black precipitates, were filtered twice and yellow brown suspension was collected. Finally, obtained GQDs were dried using rotary evaporator, and subsequently dissolved in water.

### Determination of GQDs concentration

In order to determinethe GQDs concentration, we measured absorption spectra using a UV-Vis spectrophotometer (PerkinElmer, Lambda 25). Then, according to Beer-Lambert equation, maximum absorption at 320 nm and molar extinction coefficient of GQDs (~10^6^ M^−1^ cm^−1^)^53^, GQDs concentration was determined.

### Conjugation of MPG-2H1, pDNA and GQDs

First, 1.0 µg psi-CHEK plasmid harboring firefly luciferase gene was mixed with different amounts of purified peptide in nitrogen to phosphate (N/P) ratio = 18 and incubated at room temperature for 40 min. Then, different concentrations of GQDs (180, 240, 400, 600 nM) from 800 nM stock solution were mixed with prepared pDNA/MPG-2H1 complexes. This step was performed on ice. Next, these mixtures were incubated at 4 °C under controlled agitation overnight.

### Gel retardation assay

Native acrylamide and 1% agarose gels were used to investigate the electrophoretic mobility of peptide, plasmid and complexes of pDNA/MPG-2H1 (0.1 µg pDNA and 2.43 µg MPG-2H1) and pDNA/MPG-2H1/GQDs (0.1 µg pDNA, 2.43 µg MPG-2H1, and 1.6 × 10^−2^ nmol GQDs) and followed by trypan blue staining and visualization by UV-illumination, respectively.

### GQDs characterization

UV-Vis spectrum of synthesized GQDs was measured by UV-Visible spectrophotometer (PerkinElmer, Lambda 25). Fluorescence spectra were recorded by Cytation^TM^ 3 multi-mode detection system (BioTek) at room temperature. Raman and FTIR spectra as two complementary spectroscopic techniques were recorded by Almega Thermo Nicolet Dispersive Raman spectrometer (the excitation laser was 532 nm) and Tensor 27 FT-IR spectrometer (with the KBr pellet technique), respectively. In order to determine size and height, the GQDs films were prepared by drop-casting a diluted suspension and placed on the surface of a cleaned SiO_2_/Si(100) and AFM images were taken by using an atomic force microscopy (AFM) instrument (Digital Instruments, NanoScope V). TEM images of GQDs and prepared complex were taken on lacey carbon film on copper grid by TEM, Zeiss-EM10C-80 KV. SEM image obtained using a Philips XL30 model scanning electron microscope. The ζ-potential of GQDs and prepared complexes were determined by dynamic light scattering. Zetasizer Nano ZS instrument (Malvern Instruments, UK) was used to measure ζ-potential at 4 °C. In order to determine the stability of GQDs, the PL intensity of free GQDs and GQDs in combination with MPG-2H1/pDNA complexes were investigated by Cytation^TM^ 3 multi-mode detection system at 24 h, 48 h and 72 h time intervals.

### Cell culture

HEK 293T cell line was cultured in DMEM F12 medium supplemented with streptomycin (100 mg/ml) and penicillin (100 U/ml) in 5% CO_2_ humidified atmosphere at 37 °C. Trypsin (0.25%) was used to digest the cells for 2 min, after 2 days and enable passage.

### Cytotoxicity assay

Methyl thiazolyl diphenyl-tetrazolium (MTT) reagent was used to detect the cytotoxicity of GQDs and MPG-2H1 chimeric peptide. HEK 293 T cells in their exponential growth phase were seeded in 96-well plates (2 × 10^4^ cells per well) and different concentrations of chimeric peptide (4.6, 6.9, 9.2, 11.5, 13.8, 18.4 and 27.6 µg) and GQDs (20, 40, 80, 160, 240, 320 and 400 nM) were cultured with the cells and incubated for 48 h at 37 °C in a humidified incubator with 5% CO_2_ atmosphere. 10 µl MTT salt solution (5 mg/ml) was added to each well and was further incubated for 4 h. The formazan crystals developed by vital cells were dissolved by DMSO (100 µl) and the plates were read in a microplate reader (ELx800, Biotek, USA) at 570 nm.

### Cellular bioimaging

Fluorescence microscopy technique was used to visualize the entry of biocompatible fluorescence nano-complexes into the living cells^54^. For this reason, HEK 293T cells were seeded in culture plates and incubated at 37 °C. The complexes consist of GQDs, MPG-2H1 peptide and pDNA were prepared with different concentrations as mentioned in Table 1 and incubated at 4 °C overnight. Then each one was diluted with free serum cell culture medium up to 300 µl total volume in each well. After incubation for 24 h, the fresh medium with 10% FBS was added to each well and after another 24 h the cells were washed three times with phosphate buffered saline (PBS) and finally, fluorescence imaging was done at two different wavelengths ((ex:469 nm, em:525 nm) and (ex:585 nm, em:647 nm)) by Cytation^TM^ 3 multi-mode detection system and for further investigation, cell nucleus were identified by DAPI staining (ex:405 nm, em:425–475 nm) and entry of complexes into the nucleus of the cells was conducted by confocal microscopy.

### Transfection assay

HEK 293 T cells in their exponential growth phase were seeded in 24 well plate with a density of 1 × 10^5^ per each well. In the time of transfection, the cells were 80% confluent. Two hours before transfection, serum starvation was done. Then various ratios of the already prepared GQDs/MPG-2H1 peptide/pDNA complexes (Table 1) were added to each well. After 24 h the media of the cells wasn’t removed and it was supplemented with FBS until the media of each well reached to 10% FBS, 1% Penicillin-Streptomycin and incubated for another 24 h in humidified atmosphere of 5% CO_2_ and 37 °C. PEI was used as the positive control. Firefly luciferase in Psi-CHEK plasmid was used as a reporter gene which allows rapid and quantitation. To analyze luciferase activity, the transfected cells in each well were gently washed twice with PBS and the cells were lysed with 50 µl cell culture CCLR (Cell Culture Lysis Reagent) buffer. Luciferase activity was recorded (RLU/sec) in each well in the presence of luciferin, ATP and Mg^2+^ as the firefly luciferase substrates, with a luminometer (Berthold detection systems, GmbH).

In addition, transfection was carried out in the presence of 100 µM chloroquine at the time of supplementation of cellular medium to investigate endosomal scape capacity of our complexes.

### Fluorimetric assay

Fluorimetric assay as a fast, no expensive and fairly sensitive technic was done for further assessment of transfected fluorescent nanostructures into the desired cells^55^. In this regard, HEK 293T cells in their exponential growth phase were seeded at the density of 7 × 10^4^ per each well of black 96 well cell culture plate (SPL Life Sciences). Transfection was done exactly as previously described. Before visualization with fluorescence microscope, each well was washed slowly three times by PBS. The mode of scanning area defined on BioTek’s Gen5™ software of Cytation^TM^ 3 multi-mode detection system was selected for assessment of the nanocomplexes entry into the cells.

## Electronic supplementary material

Supplementary Fig. S1.
