# Supplementary material for: Simultaneous Gene Delivery and Tracking through Preparation of Photo-Luminescent Nanoparticles Based on Graphene Quantum Dots and Chimeric Peptides
Source: Sci Rep. 2017 Aug 25;7:9552. doi: 10.1038/s41598-017-09890-y (PMC5573361; doi:10.1038/s41598-017-09890-y)
Supplement: Supplementary file 1 — Supplementary Fig. S1. [file 41598_2017_9890_MOESM1_ESM.pdf]

Simultaneous Gene Delivery and Tracking through Preparation of Photo-Luminescent Nanoparticles Based on Graphene Quantum Dots and Chimeric Peptides

Soroush Moasses Ghafary[1], Maryam Nikkhah[1], Shadie Hatamie[2], Saman Hosseinkhani[3]*

[1] Department of Nanobiothechnology, Faculty of Biological Sciences, Tarbiat Modares University, Tehran, Iran

[2] Institute for Nanoscience and Nanotechnology (INST), Sharif University of Technology, Tehran, Iran

[3] Department of Biochemistry, Faculty of Biological Sciences, Tarbiat Modares University, Tehran, Iran

Corresponding author: saman_h@modares.ac.ir

Department of Biochemistry, Faculty of Biological Sciences, Tarbiat Modares University, P. O. Box: 14115-111, Tehran, Iran

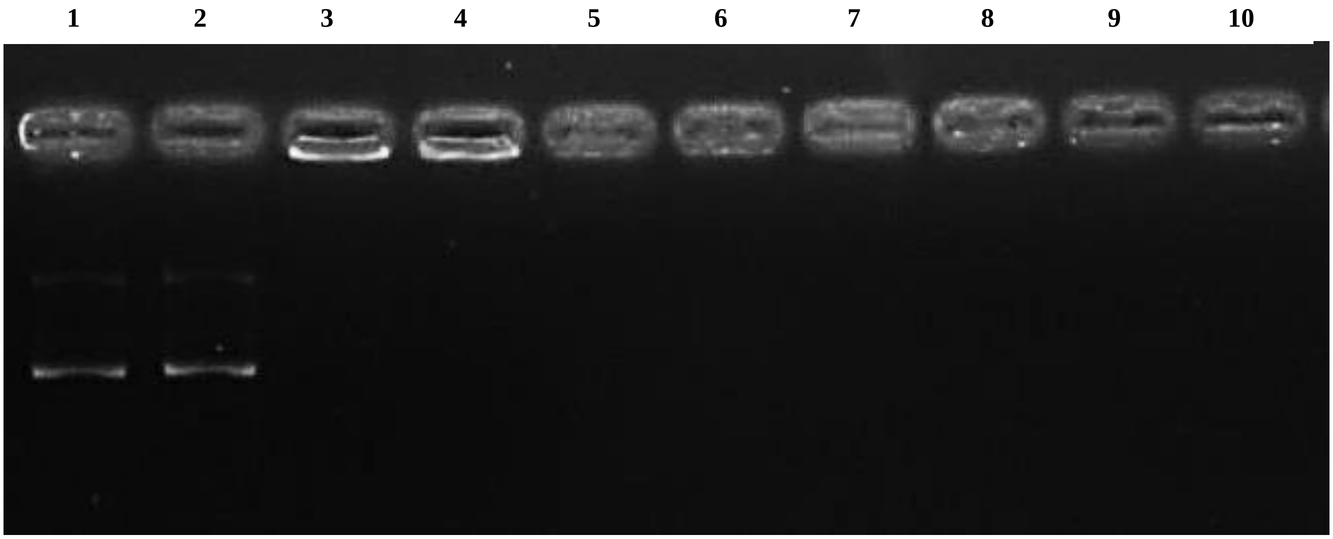

Supplementary Fig. S1. Agarose gel (1%) pDNA retardation assay. Lane 1 and its replicate lane2 are free pDNA. Lane 3 and its replicate lane 4 are MPG-2H1/pDNA complex. Lane 5 and its replicate lane 8 are complex 1. Lane 6 and its replicate lane 9 are complex 2. Lane 7 and its replicate lane 10 are complex 3.
